# ECU convention 2018 congress proceedings: Budapest, Hungary. 25-27 May 2018

**DOI:** 10.1186/s12998-018-0213-z

**Published:** 2018-09-25

## Oral Presentations

### O-01 Short or long-term treatment of spinal disability in older adults with manipulation and exercise: a randomized clinical trial

#### Michele Maiers^1^, Jan Hartvigsen^2^, Roni Evans^3^, Kristine Westrom^1^, Qi Wang^3^, Craig Schulz^3^, Gert Bronfort^3^

##### ^1^Northwestern Health Sciences University, Bloomington, Minnesota, USA; ^2^University of Southern Denmark, Odense, Denmark; ^3^University of Minnesota, Minneapolis, Minnesota, USA

###### **Correspondence:** Michele Maiers

**Study Objectives**

Back and neck pain are persistent and associated with disability and loss of independence in older adults. Spinal manipulative therapy (SMT) and supervised rehabilitative exercise (SRE) are both recommended treatments for spine pain in adults, often in combination with one another. It is unknown whether long-term management with these therapies is superior to shorter-term treatment. This study compares the effectiveness of short-term treatment (12 weeks) versus long-term management (36 weeks) of back and neck related disability in older adults using SMT combined with SRE.

**Methods and Material**

This randomized clinical trial was approved by an Institutional Review Board and conducted at a private healthcare university. Participants were 65 years of age and older, community dwelling, and self-reported both back and neck disability >12 weeks in duration. Individuals were randomly assigned to receive either 12 or 36 weeks of SMT + SRE. Co-primary outcomes were changes in Oswestry and Neck Disability Index after 36 weeks. Secondary outcomes included self-reported pain, improvement, medication use, self-efficacy, and quality of life at weeks 4, 12, 24, 36, 52 and 78. Change in objective functional measures from baseline were measured post-intervention. Linear mixed models were used to compare between group differences in an intention to treat analysis

**Results**

182 individuals participated (91 to each group). Both the short-term and long-term groups demonstrated significant improvements in back (-3.9, 95% confidence interval (CI) -5.8 to -2.0 versus -6.3, 95% CI -8.2 to -4.4) and neck disability (-7.3, 95% CI -9.1 to -5.5 versus -9.0, 95% CI = -10.8 to -7.2) after 36 weeks, with no difference between groups (back 2.4, 95% CI -0.3 to 5.1; neck 1.7, 95% CI -0.8 to 4.2). The long-term management group experienced greater improvement in neck pain at week 36, self-efficacy at week 36 and 52, functional ability and balance. On average, the short-term group attended 10 SMT and 4 SRE sessions; the long-term group attended 19 SMT and 9 SRE sessions.

**Conclusion**

For older adults with chronic back and neck disability, extending management with SMT + SRE from 12 to 36 weeks did not result in any additional important reduction in disability. Statistically significant differences in favor of long-term management were found for improvement in neck pain and self-efficacy, as well as functional measures of balance and physical performance. These findings may be important for healthy ageing and spine care in the elderly, and warrant further investigation.

### O-02 Capturing movement patterns in children using a novel 3d motion capture approach

#### Steen Harsted^1^, Anders Holsgaard-Larsen^2^, Lise Hestbæk^1,3^, Eleanor Boyle^1^, Henrik Hein Lauridsen^1^

##### ^1^Department of Sports Science and Clinical Biomechanics, University of Southern Denmark, Odense, Denmark; ^2^Department of Clinical Research, University of Southern Denmark, Odense, Denmark; ^3^Nordic Institute of Chiropractic and Clinical Biomechanics, University of Southern Denmark, Odense, Denmark

###### **Correspondence:** Steen Harsted (sharsted@health.sdu.dk)

**Background**

Studies on medial and lateral knee displacement during functional movements have until now mainly been investigated in healthy or osteoarthritic adults with regards to later injury, prevalence of pain syndromes, or progression of arthrosis. However, aberrant knee movements in children may predict musculoskeletal complications later in life. Thus, feasible and valid methods for quantifying this are needed.

The main kinematic of interest when assessing aberrant knee movement is frontal plane knee motion. This can be estimated directly in 3d (“true” knee varus), or indirectly either in 2d projections, or as ratios such as knee to hip separation distance (KHR). KHR is a measured as the distance between the knees divided by the distance between the hips.

Marker based motion capture systems are the gold standard for quantifying knee kinematics but these systems are stationary, time consuming and costly, and thus only available in very specialized settings. Contrary, markerless motion detection technology is significantly less time consuming, portable and cheaper, and the technology may have matured to the point where the accuracy of the kinematic outcome measures may be applied in health care and research.

**Methods**

We determined the concurrent validity of measuring among others the knee valgus and KHR using a portable, markerless 3D-motion capture system, “The Captury”, against a 16 camera marker-based system, Vicon, in 14 children aged 3 to 5 years.

The 14 children were recorded simultaneously by the two systems while performing a standardized series of 3 squats and 3 standing broad jumps. These movements were chosen based on their regular use in clinical practice and in motor control assessment of children.

We determined the concurrent validity by estimating among others: limits of agreement (LOA) and root mean square errors (RMSE).

**Results**

Our preliminary analyses find knee varus agreement to be low (LOA [-19.7° to 42.25°], RMSE = 6.40°) while KHR may have sufficient agreement (LOA [-0.46 to 0.39], RMSE = 0.09]) to be used in clinical practice. More results will be presented.

**Discussion**

This study provides preliminary evidence of acceptable concurrent validity of some lower extremity measurements in pre-school children made by a markerless motion capture system. This can have major implications for future evaluations of movement patterns, both in research, clinic and screening programs.

### O-03 Effectiveness of spinal manipulative therapy for chronic low back pain: results from an individual participant data meta-analysis

#### A. de Zoete^1^, S.M. Rubinstein^1^, M.R. de Boer^1^, M.W. van Tulder^1^, M. Underwood^2^, J.A. Hayden^3^, L. Buffart^4^, R. Ostelo^1^ and the Back Pain IPD consortium*

##### ^1^Department of Health Sciences, Amsterdam Public Health Research Institute, VU University De Boelelaan, Amsterdam, The Netherlands; ^2^Warwick Clinical Trials Unit, Warwick Medical School, The University of Warwick, Coventry, UK; ^3^Department of Community Health & Epidemiology, Dalhousie University, Halifax, Nova Scotia, Canada; ^4^Department of Epidemiology and Biostatistics, VU University Medical Center, Amsterdam, The Netherlands

###### **Correspondence:** A. de Zoete

*Back pain IPD consortium: G. Bronfort, N.E. Foster, C. Maher, J. Hartvigsen, P. Balthazard, F. Cecchi, M.L. Ferreira, M.R. Gudavalli, M. Haas, B. Hidalgo, M.A. Hondras, C.J. Hsieh, K. Learman, P.W. McCarthy, T. Petersen, E. Rasmussen-Barr, E. Skillgate, Y. Verma, L. Vismara, B.F. Walker, T. Xia, N. Zaproudina

**Systematic review registration:** PROSPERO CRD42015025714

**Keywords:** Low back pain, Spinal Manipulative Therapy, Individual participant data

*This abstract won 2nd Prize, but the text of the abstract is not available for publication.

### O-04 Is effectiveness of Chiropractic Maintenance Care moderated by psychological profile? A secondary analysis of a pragmatic randomized controlled trial

#### Andreas Eklund^1^, Irene Jensen^1^, Charlotte Leboeuf-Yde^2^, Alice Kongsted^3^, Iben Axén^1^

##### ^1^Karolinska Institutet, Institute of Environmental Medicine, Unit of Intervention and Implementation Research for Worker Health, Stockholm, Sweden; ^2^Institute for Regional Health Research, University of Southern Denmark, Odense, Denmark; ^3^Department of Sports Science and Clinical Biomechanics, University of Southern Denmark, Odense, Denmark

###### **Correspondence:** Andreas Eklund

**Study objectives**

The overall aim of the study was to explore the potential effect moderation of the effectiveness of Chiropractic maintenance care (MC) by psychological subgroups identified by the West Haven-Yale Multidimensional Pain Inventory (MPI).

The specific objectives were to investigate if the MPI subgroups had different outcomes in total number of days with bothersome low back pain (LBP), and the total number of visits during the study period.

**Methods and material**

This project is a secondary analysis of a pragmatic, investigator and assessor-blinded randomized controlled trial with a two-arm parallel design and 52 week follow-up period. The two treatment arms were, MC with the aim of preventing future episodes through regular visits, or control, where they were recommended to contact the clinician promptly only when symptoms returned.

At the first visit, patients were classified into one of three distinctly different psychological/behavioral subgroups, Adaptive Copers (AC), Interpersonally Distressed (ID) and Dysfunctional (DYS).

Number of days with LBP was collected weekly using an automated SMS system and number of visits were collected from the patient medical record.

**Results**

In total 252 subjects completed the trial and were included in the final analysis (Control: 162, MC: 166).

A positive effect of MC (number of days with LBP) was overserved in the DYS group (-31.6; p: 0.061; 95%CI: -64.7, 1.5) and ID group (-15.4; p: 0.568; 95%CI: -53.3, 22.5) and a negative effect of MC in the AC group (10.3; p: 0.425; 95%CI: -25.0, 45.7). When the analysis was performed with a combined ID + DYS subgroup, the effect was both large and statistically significant (-25.4; p: 0.046; 95%CI: -50.33, -0.41).

Within the AC subgroup the MC intervention resulted in a higher number of visits (3.64; p: <0.001; 95%CI: 2.0, 5.5) whereas within the ID and DYS subgroups the difference was smaller (1.71; p: <0.201; 95%CI: -0.9, 4.3 and 1.02; p: 0.588; 95%CI: -1.6, 2.9).

**Conclusion**

Patients should be considered for MC if they report high levels pain severity, marked interference with everyday life due to pain, high affective distress, low perception of life control and low activity levels and/or dysfunctional behaviors.

Patients who, on the other hand, report low pain severity, low interference with everyday life due to pain, low life distress, high activity level and high perception of life control are likely to not benefit from MC and should be recommended care only when they experience a relapse of pain.

### O-05 Motor control and musculoskeletal health in kindergarten children

#### Henrik H. Lauridsen^1^, Steen Harsted^1^, Lise Hestbæk^1,2^

##### ^1^Research Unit for Clinical Biomechanics, Department of Sports Science and Clinical Biomechanics, University of Southern Denmark, Odense, Denmark; ^2^Nordic Institute of Chiropractic and Clinical Biomechanics, University of Southern Denmark, Odense, Denmark

###### **Correspondence:** Henrik H. Lauridsen (hlauridsen@health.sdu.dk )

**Background**

It is well established that spinal pain originates early in life, and that back pain in adolescence increase the risk of similar pain significantly in adulthood. However, the age of onset of spinal pain is still unknown, and knowledge of spinal and extremity complaints and their consequences in preschool children is scarce.

Danish preschools have had an increasingly strong focus on motor skills improvements, as research suggest that motor skills are important for children’s general development. Given that inappropriate use of the musculoskeletal system may increase the risk of overuse and traumatic injuries, and that motor skills interventions have shown to decrease the risk of traumatic injuries among adolescents, a potential benefit of improved motor performance on musculoskeletal health in preschool children should be investigated. This work package is part of the MiPS study DK, and will establish five main purposes in children and adolescents aged 3 to 15 years:

- The incidence/course of back-, neck- and extremity-disorders
- Potential patterns of development of musculoskeletal disorders
- The influence of motor performance, movement patterns, strength, physical activity and parental socio-economic status in preschool on musculoskeletal health
- Normative data for movement patterns in childhood
- The predictive value of motor performance assessment and other early potential predictive markers to predict musculoskeletal health

**Methods**

We have designed a natural experiment including a cohort study. All children attending public preschools in Svendborg Municipality are invited to participate. Data from test rounds at baseline, 6, 18 and 30 months will be collected including fine and gross motor skills and movement patterns. Motor skills will be measured using the Movement Assessment Battery for Children, and movement pattern analysis will assess the drop vertical jump and the standing broad jump tests using a portable three-dimensional high-speed motion capture system (The Captury Live system). Complaints from the musculoskeletal system will be reported using bi-weekly parental SMS-track inquiring about the child’s musculoskeletal pain.

**Results**

Baseline and 6 months follow-up data has been collected on 865 children aged 3 to 5 years. We expect to present typically reported problems, including age and sex related incidence figures of the cohort at baseline.

**Discussion and conclusions**

The project will bring new insights into the debut of musculoskeletal problems, how these problems develop and the type of musculoskeletal problems children from three to 15 years of age experience. This may enable recognition of risk patterns which are important for preventing future chronic musculoskeletal conditions.

### O-06 Migraine and Tension-Type Headache in former colicky babies treated by chiropractors: a prospective cohort study

#### Jan Hoeve, Kathrine Sund

##### Chiropractie Staphorst, Staphorst, The Netherlands

###### **Correspondence:** Jan Hoeve (janhoeve@chiropractiestaphorst.nl)

**Background**

Within the headache literature recent publications point to an intimate relationship between infantile colic and later migraine [1]. A Finnish prospective cohort study of apparently untreated former colicky babies revealed 23 % migraine at age 18, versus 11 % in former non-colicky babies [2], compared to15 % in the general population [3]. In the present study we prospectively explore relationships between infantile colic and the development of adolescent migraine in a cohort of former colicky babies who at the time had been treated by chiropractors..

**Methods**

Colicky babies, who had been treated when they were less than 12 weeks old by means of a gentle chiropractic method ( J-Tech reflex instrument, zero setting and tangentially applied), were contacted some twenty years later and evaluated for migraine and tension headache. Migraine screening was performed using ID migraine [4].

**Results**

Out of a total of 442 individuals who were treated between 1993 and 2001, we managed to contact 269, 182 (68 %) boys and 87 (32 %) girls. Migraine was reported by 12 (5%), six boys and six girls, 7 (3%) had migraine with aura and 5 (2%) without aura. In 8 individuals the migraine had started at the onset of puberty, in 4 the migraine had a distinct familial relationship. In 1 almost daily chronic migraine without aura had started already by the age of 3 (Table 1). Tension type headache was reported by 42 (16%) individuals, 25 (60%) boys and 17 (40%) girls. In 12 individuals the headache had started at the onset of puberty (Table 2) .

**Discussion**

The prevalence of 5 % for migraine in our cohort of treated babies is 80% lower than the prevalence of 23% reported by Finnish researchers for a cohort of untreated former colicky babies. Underlying relationships between infantile colic and migraine are discussed.

**Conclusions**

Early chiropractic treatment directed at relieving occipital/upper-cervical dysfunction may be an effective way to prevent a migraine pattern from getting established at a very young age, thereby preventing the development of migraine later on during childhood and adolescence.

**Consent to publish**

All individuals who were contacted gave their informed consent to include the information provided in the present study and to publish the result.

**References**

[1] Gelfand, A. A, Goadsby, P. J, Allen, I. E. The relationship between migraine and infantile colic: asystematic review and meta-analysis. Cephalalgia 2015; 35(1): pp.63-72.

[2] Sillanpaa, M, Saarinen, M. Infantile colic associated with migraine: A prospective cohort study. Cephalagia 2015; 35(14): pp. 1246-51.

[3] Stovner, L.J, Andree, C. Prevalence of headache in Europe: a review for the Eurolight project. J. Headache Pain 2010; 11(4): pp. 289-99.

[4] Lipton, R.B, Dodick, D, Sadovsky, R. et al. A self-administered screener for migraine in primary care: the ID migraine validation study. Neurology 2003; 61(3): pp. 375-82.

### O-07 Patient-reported outcome measures (PROMs) in clinical practice for non-malignant pain: a realist review and theoretical framework

#### Michelle M. Holmes^1^, Felicity L. Bishop^1^, David Newell^2^, Jonathan Field^3^

##### ^1^Psychology, University of Southampton, Southampton, Hampshire, UK; ^2^AECC University College, Bournemouth, UK; ^3^Back2Health, Southsea, Hampshire, UK

###### **Correspondence:** Michelle M. Holmes (m.m.holmes@soton.ac.uk)

**Background**

The use of patient-reported outcome measures (PROMs) has increasingly been incorporated into routine chiropractic practice. Research to date suggests that PROMs may affect the process and outcome of care. The theoretical basis underpinning the use of PROMs in clinical practice remains underdeveloped; much of the published research has focused on the impact PROMs may have in clinical practice with limited research to understand the potential mechanisms behind any effects. The aim of this realist review was to identify the processes by which PROMs might influence health outcomes in routine clinical practice for non-malignant pain.

**Materials and Methods**

An electronic search was carried out of relevant databases: MEDLINE, EMBASE, PsycINFO, PsycARTICLES, Cochrane Library and Web of Science. The review examined reviews, letter, editorials and commentaries in order to identify theories and critical pieces of literature exploring how PROMs feedback might work in routine clinical practice. Text from 61 relevant papers was included and coded inductively. Codes were examined for patterns; to form a preliminary conceptual explanation of the processes and mechanisms of actions when using PROMs. Findings were reviewed in relation to formal psychological theories and empirical literature, and a theoretical framework was developed.

**Results**

The review suggests that PROMs may affect patients through various processes: incorporating increasing clinician knowledge, facilitating patient-doctor interaction, provision of patient-centered care, monitoring, informing strategies to improve care, therapeutic relationship, patient satisfaction, patient behaviour and factors which influence clinicians’ use of PROMs. The developed a novel theoretical framework: The Patient Reported Outcome Measures Pathway Theory (PROMPT).

**Conclusions**

The findings of this realist review highlight a series of processes by which PROMs may influence patient outcomes within the context of treating non-malignant pain. PROMPT provides a valuable foundation to guide future research on the use of PROMs within chiropractic care and the processes by which PROMs may influence health outcomes within the chiropractic context.

### O-08 A comparison of the effectiveness of manual therapy, exercise, and medical intervention for the reduction of subjective symptoms of dizziness in adult patients with cervicogenic dizziness: a systematic review and meta-analysis

#### Marc W. Sanders, David Newell, Johan Ramsoskar, Kim D. Kristiansen, Greg Pearse, Trym Buvarp, George Rix

##### Department of Research, AECC University College, Bournemouth, Dorset, UK

###### **Correspondence:** Marc W. Sanders (marcwsanders@gmail.com)

**Background**

Cervicogenic dizziness is diagnosed through a process of exclusion and should include symptoms of dizziness and/or disequilibrium together with neck pain or stiffness [1,2,3]. Numerous authors have suggested that a variety of manual therapy interventions can have an effect on cervicogenic dizziness [1,4,5,6]. New studies have been identified since previous systematic reviews that show additional favourable evidence.

**Objective**

To compare the effectiveness of manual therapy, exercise and medical intervention for the reduction of subjective symptoms of dizziness in patients with cervicogenic dizziness.

**Design**

A systematic review and meta-analysis of randomised (RCTs) and non-randomised controlled trials (non-RCTs).

**Methods**

The following data sources were searched, screened and extracted: Cochrane Central register of controlled trials (The Cochrane Library 2016, issue 3), MEDLINE (June 1963 to March 2016), CINAHL (October 1993 to March 2016), PEDro (up to 2016), Index to Chiropractic Literature (up to 2016) and AMED (March 1981 to March 2016). Methodological quality was assessed using the Maastricht-Amsterdam grading system and Downs and Black grading system for randomised and non-randomised controlled trials respectively. Three of the authors assessed the quality of evidence using the GRADE approach; disagreements were resolved via discussion. A meta-analysis was performed for the subjective outcome measures of Visual Analogue Scale (VAS) dizziness, Dizziness Handicap Inventory (DHI), and frequency of dizziness.

**Results**

Of the 1563 studies identified, twenty-two studies were eligible and included. Six of which were randomised controlled trials and sixteen of which were non-randomised controlled trials. A total of 1093 participants were involved across all included studies. The overall risk of bias was deemed low for all the randomised controlled trials. Of the non-RCTs assessed by the Down and Black criteria, there were no excellent papers. Two papers were graded as good, six as fair, and eight as poor. The randomised controlled trials used in the meta-analysis all showed a reduction of VAS dizziness, DHI, and frequency of dizziness across the 180 participants with a mean reduction of 25.79 (large effect), 17.38 (medium effect), and 1.26 (large effect), respectively. However, the quality of the evidence assessed using GRADE was rated as very low, low, and very low, respectively.

**Conclusions**

There is evidence to suggest that manual therapy and multi-modal therapy including exercise shows an improvement compared to other interventions including placebo for reducing VAS dizziness, DHI, and frequency of dizziness in patients with cervicogenic dizziness, however it is of a very low, low, and very low quality, respectively.

**Trial registration**

N/A

**Consent to publish**

N/A

**Acknowledgements**

We thank Professor Gordon Guyatt for his support with GRADE quality assessment.

**References**

[1] Reid SA, Rivett DA. Manual therapy treatment of cervicogenic dizziness: a systematic review. Man Ther. 2005; 10:4-13.

[2] Malmström E, Karlberg M, Melander A, Magnusson M, Moritz U. Cervicogenic dizziness - musculoskeletal findings before and after treatment and long-term outcome. Disabil Rehabil. 2007; 29:1193-1205.

[3] Hain TC. Cervicogenic causes of vertigo. Curr Opin Neurol. 2015; 28:69–73.

[4] Lystad RP, Bell G, Bonnevie-Svendsen M, Carter CV. Manual therapy with and without vestibular rehabilitation for cervicogenic dizziness: A systematic review. Chiropr Man Therap. 2011; 19:21.

[5] Reid SA, Callister R, Snodgrass SJ, Katekar MG, Rivett DA. Manual therapy for cervicogenic dizziness: Long-term outcomes of a randomised trial. Man Ther. 2015; 20:148-156.

[6] Moustafa I, Diab A, Harrison D. The effect of normalizing the sagittal cervical configuration on dizziness, neck pain, and cervicocephalic kinesthetic sensibility: a 1-year randomized controlled study. Eur J Phys Rehabil Med. 2016; 53:57-71

### O-09 How much pain reduction matters for neck pain patients undergoing chiropractic treatment?

#### B. Wirth, C. Schäfer, B.K. Humphrey, C. Peterson, P. Schweinhardt

##### Department of Chiropractic Medicine, University of Zurich/University Hospital Balgrist, Zurich, Switzerland

###### **Correspondence:** P. Schweinhardt

**Background**

Knowing the degree of pain reduction that is meaningful to patients is important to define therapeutic goals in clinical practice and treatment trials. Across different pain conditions and treatment modalities it has been shown that a two-point decrease or a 30% reduction on a numerical pain rating scale (NRS) is associated with clinically meaningful improvement (Farrar et al., 2001; Ostelo et al., 2008). However, it is unclear whether a similar pain reduction is required for neck pain patients undergoing chiropractic treatment to achieve clinically meaningful improvement. In addition, it is unknown whether this relationship depends on the time elapsed since start of treatment and/or on pain chronicity.

**Methods**

In a prospective observational study, 850 neck patients (299 male, age = 41.5 ± 13.8 years) completed the NRS before chiropractic treatment and the NRS and the Patient Global Impression of Change (PGIC) after 1 week, 1 month, 3 months, 6 months and 12 months. According to previous literature, the two highest PGIC-categories (“much better” and “better”) were defined as clinically relevant improvement. The raw and percentage NRS-changes related to clinically relevant improvement were calculated for each time point. One-way ANOVAs (post-hoc Bonferroni) were conducted to compare NRS changes (absolute and percentage) required for clinically relevant improvement at different time points and to compare percent NRS changes required for clinically relevant improvement between acute, subacute and chronic patients after 3 months.

**Results**

The percentage of improved patients increased from 55.5% after 1 week to 72.9% after 1 month, 77.8% after 3 months, 78.3% after 6 months, and 80.9% at 12 months.

NRS changes in the improved patient subgroup steadily increased with time elapsed since start of treatment up to three months: mean raw NRS changes were -3.13 (2.6) after 1 week; -3.81 (SD 2.5) after 1 month; -4.28 (SD 2.6) after 3 months; -4.36 (SD 2.5) after 6 months and -4.3 (SD 2.5) after 12 months (F(4,2488)=17.47, p<0.001). Similarly, percent changes in the improved patient subgroup increased from -47.73% (SD 55.18%) at 1 week to a maximum of just over -70%; already achieved at 3 months and stable at 6 and 12 months (F(4,2488)=24.88, p<0.001).

The mean percent NRS-changes associated with clinically relevant improvement after 3 months differed significantly between the acute patients (-79.55%, SD 33.8%), and the subacute patients (-67.14%, SD 45.2%), as well as the chronic patients (-61.95%, SD 43.5%) (F(2,522)=10.66, p<0.001; post-hoc Bonferroni: all p-values <0.02). In contrast, the percent NRS-changes of the subacute patients did not significantly differ of those of the chronic patients.

**Conclusions**

This analysis yielded three important findings: first, the raw as well as the percentage NRS changes required for clinically relevant improvement were considerable bigger than those previously described for other pain conditions and treatment modalities (raw NRS changes between 3 and 4 and percentage changes between 47% to 70% in the present study, compared to raw changes of 2 and percent changes of 30% previously reported). Second, the more time had elapsed since start of treatment, the larger the NRS changes required for clinically relevant improvement. Third, acute patients require larger NRS changes for clinically relevant improvement compared to sub-acute and chronic patients.

These results indicate that it is important to consider that the degree of pain relief required for clinically relevant improvement might depend on the type of patient group and perhaps treatment modality. This is important in order to define clear treatment goals with the individual patient.

**References**

◦ Farrar, J.T., Young, J.P., Jr., LaMoreaux, L., Werth, J.L., and Poole, R.M. (2001). Clinical importance of changes in chronic pain intensity measured on an 11-point numerical pain rating scale. Pain 94, 149-158.

◦ Ostelo, R.W., Deyo, R.A., Stratford, P., Waddell, G., Croft, P., Von Korff, M., Bouter, L.M., and de Vet, H.C. (2008). Interpreting change scores for pain and functional status in low back pain: towards international consensus regarding minimal important change. Spine (Phila Pa 1976) 33, 90-94.

### O-10 A tale of two specialities: comparing the development of radiology in chiropractic and medicine

#### Kenneth J. Young (k.young@murdoch.edu.au)

##### School of Health Professions, Murdoch University, Perth, Australia

Specialisation in medicine has been notably successful, with advanced training and enhanced capabilities in specialised skills leading to better outcomes for patients and increased prestige for practitioners. Complementary and alternative medicine (CAM) professions have been less successful in this endeavour. Specialist radiology training in chiropractic bears a strong resemblance to that of medicine, with the eventual development of competitive entry for ‘residencies,’ rigorous certification exams, and the creation of a journal as well as specialist professional organisations. Yet radiology has not been fully recognised as a speciality in chiropractic, whereas in medicine it has flourished. Reasons include incomplete acceptance of the biomedical model by chiropractors, low numbers of ‘chiropractic radiologists,’ and resistance by external groups. This paper uses radiology as a case study of the forces influencing the failure of development of a speciality in a CAM profession with comparison to its successful analogue in medicine.

**References**

There are about 100 references in this paper, available upon request; they include books, peer-reviewed journal articles, other publications, personal communications, and interviews.

### O-11 Chiropractic care as a richly contextual package: contemporary challenges and opportunities in undergraduate curriculum

#### David Byfield, Alister DuRose, David Newell

##### Faculty of Life Sciences and Education, University of South Wales, Treforest, UK

###### **Correspondence:** David Byfield

To maintain credibility in a modern healthcare environment, undergraduate health educational programmes must be evidence based reflecting best evidence and practice within the curriculum in order that new graduates are well prepared for professional life the possessing skill sets to meet the demands of clinical practice. As new clinical science emerges pertaining to the delivery of best care and explanations underpinning clinical outcomes, chiropractic curricula need to be reviewed and updated in terms of content and delivery in order to remain contemporary and to comply with current guidelines and statutory requirements. However, embedding new concepts and content in an already busy programme requires considerable time and resources from educational institutions. Creating new themes within an existing programme, both vertically and horizontally poses a challenge for academic staff to ensure that the knowledge and skills are integrated, delivered and assessed appropriately in line with other units of delivery. A good example of this situation is the current clinical evidence pertaining to the role of contextual factors and their impact on enhancing patient outcomes, particularly in relation to care and management of musculoskeletal conditions. This presentation will outline some of the opportunities and barriers associated with introducing this important evidential paradigm into an existing traditional chiropractic undergraduate curriculum. The aim of this presentation is to launch a discussion of the importance of these factors within the delivery of effective health care and how this knowledge and skill can be packaged to improve and enhance student understanding of their role as a primary contact professional. The presentation will also address how knowledge of and enhancing of these factors can align with and enhance traditional historical mind sets regarding chiropractic practice in creating a small but influential paradigm shift in undergraduate education.

## Poster Presentations

### P-03 The relationship between Video Enhanced Observations and VIVA examination performance in second year chiropractic undergraduate students: a cross sectional cohort study

#### Danny Clegg, Alister DuRose, David Byfield

##### Faculty of Life Sciences and Education, University of South Wales, Pontypridd, UK

###### **Correspondence:** Danny Clegg

**Introduction**

Manipulative psychomotor skills training forms a fundamental part of undergraduate Chiropractic education. As new learning techniques and strategies are developed, it is important to be able to quantify their effectiveness. Video Enhanced Observation (VEO) is a server-based software, which allows for pre-programming of digital “tagsets” as well as the ability to upload, tag, and digitally comment on video recordings of various manual procedures. The aim of this study was to investigate relationships between immersive learning using VEO in Chiropractic technique skills training, and VIVA performance scores.

**Methods**

VEO was integrated into the year 2 Chiropractic technique module at the Welsh Institute of Chiropractic (WiOC) during the 2017-18 academic year. VEO recordings were collected, tagged and uploaded to a secure server. Once uploaded the videos were shared with peers and academic members of staff. All 58 active year 2 students were enrolled and given creative access to the system so that they could either: be recorded during a class with verbal and digital feedback given immediately afterwards; be recorded during class with digital tags and written feedback applied at a later stage; or upload their own recorded technique performance to the server for feedback from the VEO manager. Students also had access to a library of technique exemplars as performed by an experienced Chiropractic educator. The total number of occasions a student was involved in a video was recorded, with data further divided into 2 groups for comparison: number of times a student was recorded by a tutor during class; number of videos recorded and uploaded to VEO by the student. All variables were analysed for correlations with technique VIVA percentage scores. Data were analysed for normality and appropriate correlation tests were performed using SPSS V.24 for Windows.

**Results**

31 students (53%) participated in classroom recordings. Of those 31 students, 7 (12%) recorded and uploaded at least one video for digital feedback. There was positive correlation between the number of recordings students were involved in and the VIVA scores (r = 0.273, p=0.038). Correlations were also found when comparing those who uploaded their own videos with VIVA scores (r =0.257, p=0.052). There was a non-significant positive correlation between those solely being recorded in the classroom and VIVA scores (r = 0.158, p=0.238).

**Conclusion**

This initial study demonstrated that students participating in the VEO learning environment scored higher in the VIVA examination, indicating a more complete acquisition of the required skills and competencies associated with the assessment point. This study also indicates that the VEO system may be a valuable teaching aid for students learning complex psychomotor skills at undergraduate level.

### P-04 The “Hydrapractic” Initiative: innovative and immersive learning for chiropractic students, using police and emergency services training tools

#### Danny Clegg, Dean Whitcombe

##### Faculty of Life Sciences and Education, University of South Wales, Pontypridd, UK

###### **Correspondence:** Danny Clegg

**Introduction**

The Hydra Minerva immersive learning system is a computer simulation learning environment that enables the monitoring of real-time leadership and decision making during critical incidents. Historically it is used for the training of police and emergency services.

Hydra allows for multiple groups to be simultaneously presented with information and tasks for completion. Segregation of groups ensures smaller group sizes, increased participation, and the ability to compare and contrast group responses in a debriefing plenary room after all tasks are completed.

Chiropractic students have limited opportunities to experience real patient interactions and critical decision making before they begin their clinical training. “Hydrapractic” was designed as an initiative to develop Chiropractic learning processes using the Hydra system. This article details the inaugural Hydrapractic sessions at the University of South Wales, and explores possible applications for its future use.

**Methods**

A series of simulated, videoed (with audio) patient scenarios were written, filmed, directed, edited and acted by the lead author, with the assistance of a student clinician. The videos were further trimmed, uploaded and coordinated through Hydra Minerva. Scenarios were loaded and then timed tasks would appear for students to complete in groups of 5-6 people. Participants were asked to complete evaluation forms after each session.

The session ran twice in one day to accommodate a cohort of 59 students, with 49 students attending in total. Sessions were comprised 4 dedicated group pods, plenary room for briefing and debriefing, and the Hydra Minerva control room. The session covered scenarios from the patient follow-up visit, a pre-recorded voicemail, a telephone call between clinician and patient, a final “reveal” physical sign indicating severe pathology, and various examples of good and bad practice. A total of 6 videos and 6 tasks, lasting approximately 80 minutes with 60 minutes for debrief in the plenary room.

**Results**

49 of 59 year 3 students attended the session using Hydra facilities. 17 students completed the feedback questionnaire.

Positive feedback themes taken from the questionnaire centred around the:

- Positive Learning experience
- Desire for the technology to be used on other modules
- Contextualising and applying knowledge to patient situations prior to clinical year
- Improving student satisfaction

Negative feedback themes:

- Audio quality was poor at times
- Students wished they had been given this type of learning experience throughout earlier stages of their education
- Time allowed to complete tasks wasn’t always perfect

**Conclusion**

Hydra Minerva technology and protocols can be adapted to curriculum(s) other than police and emergency services training. Simulated scenarios can be created to suit any module or content, and the process is adaptable using other similar methods. The feedback from the inaugural Hydrapractic sessions will inform the design of future scenarios with a more structured method for deployment and execution.

### P-05 Since Panjabi’s seminal 1992 papers, how have studies that combine lumbar kinematic and muscle activity measurements improved understanding of sub-system interactions? A systematic review

#### Alister du Rose¹^,^²

##### ¹Faculty of Life Sciences and Education, University of South Wales, Pontypridd, UK; ²Centre for Biomechanics Research, AECC University College, Bournemouth, UK

**Background**

Spinal stability was interpreted by Panjabi (1992) to be dependent on co-ordinated interactions between three sub-systems, the passive, the active, and the neural control systems. If there is dysfunction within a specific system, compensation may be provided by adaptations in another [1]. To improve understanding of these interactions, it is necessary to take an approach that incorporates the concurrent measurement of several systems [2], requiring instrumentation that can do so dynamically. Physical activities involving sagittal bending are commonplace activities of daily living [3], and so an improved knowledge of sub-system interaction during lumbar flexion would be of clinical interest. To the author’s knowledge, there is currently no review of studies that have investigated dynamic flexion movements using a combination of EMG and lumbar kinematic measurements. As such it is not clear how understanding of Panjabi’s spinal stability concepts has advanced with regards to this functional movement of the spine.

**Methods**

Pubmed and Cochrane databases were searched in March 2017. The search was performed using combinations of the following keywords: Electromyography (EMG), Flexion Relaxation (FR), Kinematics, Range of Motion, Low Back Pain (LBP), Lumbar Spine, Flexion, Bending, Stability and Stabilisation. Articles had to incorporate the concurrent *in vivo* investigation of lumbar kinematic and EMG variables during dynamic lumbar flexion to be included. The review adhered to the PRISMA guidelines [4], and used a quality assessment tool developed by Abboud et al. [5]. Abboud et al. 2017 also created an assessment to interpret the quality of studies incorporating EMG, based on SENIAM guidelines [6]. This novel assessment was also incorporated.

**Results**

Out of a total of 736 articles identified through the literature search only 21 satisfied the inclusion/exclusion criteria. The screening process is outlined in the PRISMA flowchart. The combined overall and EMG quality index scores ranged from 47-100% with a mean total score of 77%. All studies could be placed in one of 4 categories, FR, comparison between LBP and controls groups, EMG activation and spinal modelling. The studies generally supported the notion that increased muscle activity is a mechanism that increases spinal stability, and that it is possible to distinguish between LBP and healthy control groups using kinematic and EMG variables.

**Conclusions**

Whilst no reviewed study specifically explored sub-system interaction, many related their findings to such mechanisms. A common weakness in study design was the use of regional measurements, which can only ever provide a broad interpretation of sub-system interaction. In addition sample sizes were typically small, and there was no consistency in methodology. Until one can measure *in vivo* inter-vertebral dynamic kinematics and relate it to one of the other sub-systems in detail, it will not be possible to make significant progress in this area. This lack of progression was reflected in this review, and highlights the requirement for new approaches to research that incorporate these elements.

**References**

[1] Panjabi, M.M., The stabilising system of the spine - Part 1: Function, dysfunction, adaptation and enhancement. Journal of Spinal Disorders, 1992. **5**(4): p. 383-389.

[2] Dankaerts, W., O'Sullivan, P.B., Burnett, A.F., Straker, L.M., Davey, P., Gupta, R., Discriminating Healthy Controls and Two Clinical Subgroups of Nonspecific Chronic Low Back Pain Patients Using Trunk Muscle Activation and Lumbosacral Kinematics of Postures and Movements. Spine, 2009. 34(15): p. 1610-1618.

[3] Colloca, C.J. and R.N. Hinrichs, The biomechanical and clinical significance of the lumbar erector spinae flexion-relaxation phenomenon: A review of literature. Journal of Manipulative and Physiological Therapeutics, 2005. 28(8): p. 623-631.

[4] Moher, D., Liberati, A., Tetzlaff, J., Altman, D.G., for the PRISMA Group, Preferred reporting items for systematic reviews and meta-analyses: the PRISMA statement. British Medical Journal, 2009. 339: p. 332-336.

[5] Abboud, J., et al., Effects of Muscle Fatigue, Creep, and Musculoskeletal Pain on Neuromuscular Responses to Unexpected Perturbation of the Trunk: A Systematic Review. Frontiers in Human Neuroscience, 2017. 10.

[6] Hermens, H.J., et al., European Recommendations for Surface Electromyography. Results of the SENIAM project. 1999.

### P-06 Motor skills in PreSchool (MiPS): study protocol for a cohort study of Danish preschool children with a nested RCT

#### Lise Hestbaek^**1,2**^, Sarah T. Jensen^1^, Thomas Skovgaard^1^, Line G. Olesen^1^, Mette Elmose^3^, Dorthe Bleses^4^, Simon C. Andersen^4^, Henrik H. Lauridsen^1^

##### ^1^Nordic Institute of Chiropractic and Clinical Biomechanics, University of Southern Denmark, Odense, Denmark; ^2^Department of Sports Science and Clinical Biomechanics, University of Southern Denmark, Odense, Denmark; ^3^Institute of Psychology, University of Southern Denmark, Odense, Denmark; ^4^TrygFonden's Centre for Child Research, Department of Economics and Business Economics, Aarhus University, Aarhus, Denmark

###### **Correspondence:** Lise Hestbaek

**Background**

Good motor skills are considered important for children’s physical, social and psychological development, but the relationship is still poorly understood. The preschool age seems to be decisive for the development of motor skills and probably the most promising time-window in relation to preventive strategies based on improved motor skills. This research program has four overall aims: 1) investigation of the effect of a structured program to improve motor skills in 3-6-year old children on current and future motor skills, musculoskeletal health, general health, cognition, and wellbeing; 2) establish reference data on motor skills in 3-6-year olds; 3) description of early development of musculoskeletal problems; and 4) establishment of a population-based cohort with inception at the age of three to six.

**Methods**

Beginning in January 2017, a new program aimed at optimizing the children’s motor skills will be implemented gradually over a four-year period in all preschools in a Danish municipality. Prior to this, a cohort was established, inviting all 1461 children attending the preschools by August 2016. Physical and cognitive measurements (including movement patterns) will be repeated yearly as long as the children attend preschool and on a smaller scale throughout the school years; musculoskeletal pain will be monitored with biweekly SMS-questions answered by the parents until the child leaves school; general wellbeing and language development will be assessed by preschool staff during preschool and by self-report later; academic performance and health care usage will be retrieved from registers.

By introducing the program into a subset of the preschools at onset and compare these children to another subset (control) which will not receive the intervention the first three years, it is possible to document a potential effect of the intervention. Seventeen preschools (including 727 children) participate in this nested RCT.

**Results**

Children were recruited to the cohort from August 2016 till the end of 2017. However, inclusion into the RCT was terminated in January 2017 by the completion of baseline data collection. The parents of 865 children (59%) have agreed to participate in the study, of which 485 attend preschools included in the RCT (67% of the eligible RCT-children).

**Discussion**

The cohort will provide important information about normal motor skills, early development of musculoskeletal problems, and the influence of early motor skills on children’s development across many domains and their potential interactions.

**Trial registration:** ISRCTN23701994.

### P-07 Chiropractors’ views on the use of patient-reported outcome measures in clinical practice

#### Michelle M. Holmes^1^, Felicity L. Bishop^1^, David Newell^2^, Jonathan Field^3^

##### ^1^Psychology, University of Southampton, Southampton, Hampshire, UK; ^2^AECC University College, Bournemouth, UK; ^3^Back2Health, Southsea, Hampshire, UK

###### **Correspondence:** Michelle M. Holmes

**Background**

Patient-reported outcome measures (PROMs) are widely available for use in musculoskeletal care. However, there is little research exploring the implementation of PROMs. This qualitative study explored chiropractors’ views on PROMs to identify any barriers and facilitators to implementing PROMs in chiropractic care and the training needs of chiropractors regarding the use of PROMs.

**Materials and Methods**

Contact was made with chiropractors working in chiropractic companies with multiple clinic sites. Semi-structured interviews were conducted with eight chiropractors, either face-to-face at their place of work or over the telephone. The interviews were transcribed and analysed using thematic analysis.

**Results**

Chiropractors discussed their knowledge and engagement with PROMs in clinical practice, identifying reasons for their use. They also discussed how they used PROMs within their clinical practice and the benefits of using them with individual patients. Chiropractors voiced concerns about patient engagement with PROMs and the appropriate PROMs to use with patients with pain. Finally, chiropractors acknowledged the environmental barriers and facilitators to using PROMs within their practice.

**Conclusions**

Using chiropractors’ views of PROMs, the study identified barriers and facilitators to implementing PROMs in chiropractic care, such as clinician knowledge, engagement, and environmental barriers and facilitators; identify the potential training needs of chiropractors regarding PROMs. The results from the study suggested chiropractors use PROMs with their individual patients, but PROMs should be meaningful to patients and chiropractors to improve engagement.

### P-08 Back Complaints in Elders (BACE): a prospective, longitudinal analysis of older people with low-back pain in chiropractic care

#### Alan Jenks,^1^ Sidney Rubinstein^1^, Iben Axen^2^, Jonathan Field^3^, David Newell^4^, Trynke Hoekstra^1^, Jan Hartvigsen^5^, Bart Koes^6^ , Maurits Van Tulder^1^ And The Bace Consortium

##### ^1^Department Of Health Sciences, Faculty Of Science, Vrije Universiteit Amsterdam, Amsterdam, The Netherlands; ^2^Department Of Intervention And Implementation Research, Institutet Of Environmental Medicine, Karolinska Institutet, Stockholm, Sweden; ^3^Private Practice, Hampshire, UK; ^4^AECC, Bournemouth, UK; ^5^Department Of Sports Science And Clinical Biomechanics, University Of Southern Denmark, Odense, Denmark; Nordic Institute Of Chiropractic And Clinical Biomechanics, Odense, Denmark; ^6^Department Of General Practice, Erasmus University Medical Center, Rotterdam, Netherlands

**Background**

Low-back pain is the most important non-fatal disease in Europe and is associated with increasing healthcare costs. These costs are likely to become greater as the population becomes older. Low-back pain, particularly in older people, results in reduced quality of life, reduced social participation and increased isolation, as well as being associated with comorbidities.

One conservative treatment for the treatment of low-back pain which is safe andeffective is spinal manipulative therapy. In contrast to their younger counterparts, relatively little investigation has been conducted amongst older people with low-back pain in a chiropractic setting.

**Aim**

This study would provide the necessary knowledge to improve our understanding for this patient population in order to provide safer and more effective care.

**Study design and population**

A prospective, multi-center practice-based cohort study will be used to collect data from older patients (>55 years of age) with low-back pain who visit a chiropractor with a new episode of low-back pain. Participants are to be recruited from the private practices of chiropractors in The Netherlands, Sweden, and the UK. Treatment will

**Outcome measures**

The following primary outcomes are to be measured using self-report, validated questionnaires: 1) pain intensity (11-point VAS), 2) low-back pain-specific functional status (Oswestry Disability Index), 3) self-perceived recovery (7-point Likert scale), and 4) EQ-5D-5L. Follow-up is to be conducted at the end of the second visit, and at 6 weeks and at 3, 6, 9 and 12 months.

**Implications of this project**

This project is modelled after the BACE study (BAck Complaints in Elders), which is currently being conducted in primary care in The Netherlands, Australia and Brazil. BACE is supported by an international consortium consisting of world-leaders in research of low-back pain. Aligning ourselves with this consortium represents a unique chance for chiropractic.

### P-09 An initial investigation of the use and understanding of the terms

#### Troy Magowan, Peter McCarthy

##### Welsh Institute of Chiropractic, University of South Wales, Pontypridd, UK

**Intro**

Certain terms used by chiropractors to define their own, or others, practice methodology have also had the unfortunate consequence of creating potential schisms in the profession. Such a situation can be exacerbated by a lack of clear accepted definitions; therefore, it is important to define such terms appropriately. Terminology that fits this category includes those words related to treatment protocol methodology for the patient who has achieved or is close to full recovery. The aim of this study was to consider use and definition of the terms Maintenance and Wellness.

**Study objectives**

To uncover the current perception of UK chiropractors regarding the meaning of the terms “Wellness care” and “Maintenance care” and how these methodologies are integrated into clinical practice.

**Methods**

A mixed methods study was used, involving a questionnaire comprised of both closed multiple choice questions and qualitative style open ended questions was created using the website SurveyMonkey. The questionnaire was then distributed electronically to 1225 Royal College of Chiropractors members and made available for 2 weeks. The chiropractic undergraduate research module ethics group at University of South Wales approved this study.

**Results**

128 completed questionnaires were received (10% of surveyed population). Of these 4% reported using a strict Wellness care model, 49% used maintenance care only and 43% reported using a combination of both in their practice.

Regarding definition of the terminology: Wellness care was perceived as treatment aimed at asymptomatic patients with the objective of “optimising body function”. Whereas maintenance care was considered to target supporting a symptomatic or chronic pain patient by maintaining the improvements achieved during the initial treatment period.

There was some agreement on both the aims of the treatment as well as the frequency. However, a number of responses also represented strongly polarised remarks. These remarks highlighted the large emotional component associated with the use of these terms by a small proportion of the population responding to this survey.

**Conclusion**

This data shows that there is some consensus among UK chiropractors regarding their understanding of the terms Maintenance care and Wellness care. Unexpectedly, a large proportion of those responding used both methodologies. This study produced evidence in support of both the need for, and importance of clearly defining the terminology used by chiropractors from the perspective of uniting the majority and reducing the emphasis of the vocal minority.

### P-10 Incidence of intersegmental cervical joint motion dysfunction in patients with Tinnitus as their co-morbidity

#### Peter McCarthy, Manuel Cabrera

##### School of Health, Sport and Professional Practice, Welsh Institute of Chiropractic, University of South Wales, Pontypridd, UK

###### **Correspondence:** Peter McCarthy

Determining whether manual palpation can reveal diagnostically relevant information about visceral disease has been subject to much debate. Although neurologically plausible, reliability of palpation has been questioned.

Tinnitus refers to the perception of a sound not being generated by an external source. The term tinnitus derives from the Latin Word *tinnire*, meaning to ring. Accumulated evidences suggest that tinnitus-related neural activity is more complex and multimodal than previously thought, however, where there is sensory input there should be some resulting change in motor output. Indeed, a relationship has been reported between tinnitus and Cervical Muscle Tenderness, TMJ Dysfunction even myofascial trigger points in the neck musculature.

***Objective***: to determine if there is a relationship between the prevalence of intersegmental joint restriction across the Cervical spine and the presence of Tinnitus (as a co-morbidity).

***Methodology***: A retrospective study compared the cervical spinal restrictions as mapped and noted in Welsh Institute of Chiropractic (WIOC) Clinic files by the Senior Student Clinicians between patients complaining tinnitus as a co-morbidity and those that did not. Files were included only if the patient had signed consent for their information to be included in such anonymised, secondary data analysis; had reported cervical palpation and either had or did not have tinnitus as a co-morbidity. Approval was granted by the chiropractic undergraduate research ethics subgroup. Data was coded in relation to the presence or absence of noted restriction in intersegmental motion. Chi squared analysis was used to compare groups.

***Results***: A total of 1537 files were searched generating 103 with tinnitus; of these 23 were discarded (incomplete) resulting in inclusion of 80. Random sampling from within the non-tinnitus population produced a control group of similar size (n=80). A statistically significant increase in noted restricted motion was found in the test group across the segments C2/3 (57.5% and 76.9%, control and test respectively: p=0.038).

***Conclusions/Recommendations:*** It may be possible to use this approach to detect differences in segmental motion, which relate to the presence of neurologically linked to sensory input from dysfunctional organs.

### P-11 A novel approach to improving breastfeeding rates and enhancing clinical education: a mixed-methods investigation of a student-led interprofessional breastfeeding clinic

#### Amy Miller

##### AECC University College, Bournemouth, UK

The importance of breastfeeding for the health of the mother and infant are clear. The World Health Organization (WHO) recommends exclusive breastfeeding for the first six months of life, with continuation of breastfeeding alongside solid foods until two years or beyond. Despite best efforts, only 1% of infants are exclusively breastfed at six months of age in the UK. In order to provide support to mother-infant dyads to breastfeed, and to provide interprofessional clinical experience to students, Bournemouth University and AECC University College created a student-led interprofessional breastfeeding clinic (ISLBC), run by midwifery students and chiropractic interns. Interprofessional education is supported and recommended by the WHO as a means to develop a collaborative practice ready workforce, and student-led clinics are one way to provide interprofessional education.

There is some low-level evidence that chiropractic care supports breastfeeding. It has been noted that intervention at birth is associated with breastfeeding difficulties and premature cessation. Intervention at birth has been suggested as a mechanism of injury in the neonate, negatively impacting on feeding mechanisms and biomechanics. Additionally, there is some evidence for an interprofessional midwifery and chiropractic intervention in preserving breastfeeding in dyads who are at risk of early cessation with multiple breastfeeding problems. However the research to date is not definitive.

**Objectives**

1. To compare breastfeeding outcomes and experiences of mothers who attend a student-led interprofessional breastfeeding clinic with mothers who receive routine care
2. To explore a student-led interprofessional breastfeeding clinic as a means of developing students’ clinical knowledge and skills, and supporting interprofessional education.

**Methods**

The first objective will be assessed using a 2-arm prospective cohort study measuring feeding outcomes at six and twelve weeks of age, one group recruited from the interprofessional student-led breastfeeding clinic, the other group will have no intervention beyond their routine care. Interviews with a sample of the mothers in the prospective study will explore the maternal experiences of breastfeeding. The second objective will be explored via focus groups with the students involved in the clinic, and focus on their learning in this setting.

**Impact**

Results from this study will be used to inform future use of this clinic, and future research. This study will be used to clarify the role of chiropractic care within the wider team supporting breastfeeding, which is a crucial public health issue facing infants in the UK.

### P-12 Is there an association between birth type and area of musculoskeletal complaint in the neonate?

#### Amy Miller

##### AECC University College, Bournemouth, UK

**Introduction**

Increasingly, parents present their infants to the chiropractor. Throughout the chiropractic paediatric literature, certain demographics are persistently different to the general population. Intervention during birth is one factor which is commonly over-represented in chiropractic paediatric populations, and has been used to describe the mechanism of injury in this age group. Birth intervention has been associated with cranial deformation, torticollis and breastfeeding difficulties. The aim of this study is to gain a more detailed understanding of specific birth interventions in infants with specific musculoskeletal problems, and identify any association.

**Methods**

A cross-sectional study utilised chiropractic intern report of specific birth type, feeding difficulties and musculoskeletal complaints in a cohort of infants presented to an interprofessional breastfeeding clinic. Descriptive data and risk ratios were used to highlight common presentations and determine associations between birth type and area of musculoskeletal involvement.

**Results**

In this cohort of 301 infants presented to an interprofessional clinic with breastfeeding difficulties, 83% had intervention at birth. The most common feeding complaints were difficult attachment, pain during feeding, and unilateral feeding preference. The most common areas of musculoskeletal involvement were thoracic, cervical and SCM.

Any type of assistance at birth increased the risk of baby being unsettled at the breast by 1.62. Induction increased the risk of cervical spine involvement by 1.86 compared to unassisted delivery. Emergency Caesarean deliveries had a 1.71 increased risk of unsettled feeding and cervical spine involvement compared to unassisted deliveries. Ventouse delivery increased the risk of thoracic spine involvement by 1.34, and unsettled feeding by 1.38, compared to unassisted delivery. Forceps deliveries increased the risk of difficulty attaching to the breast by 1.49, and cervical spine involvement by 1.30.

**Discussion**

This study is the first to our knowledge which highlights specific birth interventions as risks for specific musculoskeletal problems. Given these musculoskeletal problems, it may be appropriate that chiropractic care forms part of the infants’ healthcare. The sample size is relatively small, the study is observational, and relied on intern report.

**Conclusion**

There are significant limitations to this study. However, it provides a starting point in a previously unexplored area and hence has implications for further research. Infants who undergo assisted birth experience multiple feeding difficulties and multiple areas of musculoskeletal complaint. Forceps and emergency Caesarean deliveries appear to pose additional risk, and it may be appropriate that chiropractic is part of routine assessment for these infants.

### P-13 Self-reports of spinal stiffness compared to physical measures of spinal stiffness

#### Jones Nielsen, Casper Nim, Søren O'Neill, Jan Hartvigsen, Greg Kawchuk

##### University of Southern Denmark, Department of Sports Science and Clinical Biomechanics, Odense, Denmark

###### **Correspondence:** Jones Nielsen

**Study objectives**

1) Examine the association between scores of the Lumbar Stiffness Disability Index (LSDI) questionnaire and objective, physical measures of stiffness at the L3 segmental level in patients with persistent non-specific low back pain (nsLBP); 2) Assess if physical measures of stiffness in the lower back in these patients change following a series of spinal manipulation therapy (SMT) over 2 weeks.

**Methods and material**

15 participants were recruited from a multidisciplinary Spine Center located in Middelfart, Denmark, after being thoroughly examined by a clinician specialized in spine disorders and diagnosed with persistent nsLBP. All participants received spinal stiffness testing as well as completing the LSDI-questionnaire at baseline and at follow-up after 2 weeks. All participants received four sessions of SMT at the Spine Center. Spearman’s rank correlation was used to examine the association between the LSDI-scores and measured stiffness. A paired-samples t-test was used to determine differences in stiffness.

**Results**

We found a moderate negative correlation between the LSDI-score and the Global Stiffness of L3, *r*_*s*_ (13) = -0,567, *p* < 0,05 ( *p* = 0,027). Participants were less stiff at follow-up compared to baseline in the L3 segment following SMT (4,652 ± 0,720 N/mm versus 4,877 ± 0,858 N/mm), although the mean difference of -0,224 (95% CI, -0,837 to 0,390) N/mm was not statistically significant, *p* = 0,447.

**Conclusion**

A negative association was found between the LSDI-scores and the measured stiffness at the L3 segment. A reduction in spinal stiffness was found following spinal manipulation over a 2-week period, however the difference was not statistically significant. These results are preliminary and the results may change, since more participants will be included. At the next round of data analysis, outcomes of another questionnaire (the Lumbar Spine Instability Questionnaire) will be added and correlated to measures of stiffness to gain further insight in subjective notions of spinal stiffness. Additional lumbar levels and different types of measured stiffness will be analysed as well.

### P-14 Effects of chiropractic manipulation on subchondral bone status, cartilage and synovial membrane in an experimental model of osteoarthritis in rabbits

#### Arantxa Ortega-De Mues^**1**^, Francisco Miguel Conesa^2^, Ricardo Fujikawa^1^, Arancha Mediero^2^, Paula Gratal^2^, Francisca Mulero^3^, Gabriel Herrero-Beaumont^2^, Raquel Largo^3^

##### ^1^Madrid College of Chiropractic-RCUEMC, Madrid, Spain; ^2^IIS-Fundación Jiménez Díaz-UAM, Madrid, Spain; ^3^Centro Nacional de Investigaciones Oncológicas (CNIO), Madrid, Spain

###### **Correspondence:** Arantxa Ortega-De Mues

This abstract is not included here as it has already been published [1].

Reference

[1] Conesa-Buendía FM, Fujikawa R, Mediero A, Gratal P, Mulero F, Ortega-De Mues A. Changes in subchondral bone status, cartilage and synovial membrane in response to chiropractic manipulation in an osteoarthritis model. Osteoarthritis and Cartilage. 2018 Apr 1;26:S319.

### P-15 The use of Facebook as a formative peer assessment tool

#### Jacqueline Rix

##### AECC University College, Bournemouth, UK

**Introduction**

In healthcare teaching institutions, students are required to learn a number of practical diagnostic and treatment tasks. These tasks are psychomotor rich skills, each with a theoretical and cognitive component. In order for students to learn a psychomotor task, feedback is desired. This study aims to investigate a different method of supplying the student with feedback through the use of Facebook as a platform for peer-feedback and discussion.

**Methods**

The study was a mixed method study. All students enrolled onto year one MChiro at the AECC University College were invited to participate in the study. The first 13 students (10% of the cohort) to email their interest were enrolled onto this pilot study. Pedagogical support for participants was given and a Facebook invitation to the private Facebook Group was sent. Participants were encouraged to post videos of themselves performing psychomotor tasks to gain feedback from their peers, as well as to post research articles, pictures or questions regarding pathologies appropriate to a first year level. In semester one, the researcher was an equal partner in Facebook participation. In semester two, the researcher withdrew from participating, but remained as an observer. At the end of each semester, participants were given a questionnaire to complete and a follow up semi-structured interview was done with questions based on Facebook participation. Participant summative assessment results were compared to Facebook Group participation.

**Results**

Eleven participants completed the study. The average age was 26.5 (9.7) which was significantly different to the remaining cohort. In semester 1, 55% of participants posted videos; 100% of participants received feedback. In semester 2, 45% of participants posted videos; 100% of participants received feedback. 100% of students found the Facebook page useful. Students in the study had significantly better summative marks in reflective essay writing, however were equal with the remaining cohort in the theory assessment. Participants who participated in the Facebook page significantly outperformed participants who did not participate in the Facebook page in the semester two practical assessment.

**Conclusion**

Students did use the Facebook Group and found it useful, participation was low and it was seen by some students as an additional chore, rather than the learning environment it was intended to be. That being said, students may not have used it in the way the researcher envisioned, but reported that they did learn from the FB group and would use it again in future.

### P-16 Comparison of HVLA lumbosacral manipulation and sham manipulation on running time and horizontal jump among amateur soccer players

#### Resat Coskun^1^, Bülent Aksoy^2^, Doruk Turhan^3^, Mehmet Toprak^2^

##### ^1^Arel University, Istanbul, Turkey; ^2^Bahcesehir University, Istanbul, Turkey; ^3^Altinbas University, Istanbul, Turkey

###### **Correspondence:** Resat Coskun (resatcoskun@arel.edu.tr)

It was aimed to investigate the effects of high velocity low amplitude (HVLA) manipulation, sacroiliac and lumbosacral manipulation on sprint, hurdle race and jumping performance among amateur soccer players who were diagnosed with asymptomatic dysfunctions in sacroiliac and lumbosacral joints.

Before and after application, 20 meters of sprint and 20 meters of hurdle race time and horizontal jump distance were measured. We measured sprint and hurdle race times with a timer and video recordings. 30 patients were included in the study. We divided them in two groups as 15 individuals in each group and made a random selection of patients. One-time sham manipulation applied to the control group while one-time chiropractic HVLA lumbosacral manipulation applied to the experiment group.

20-meter sprint score in control group decreased from 3.49 seconds to 3.46 seconds. Total variation is 0.03 sec. In the experimental group, the 20-meter sprint score decreased from 3.44 seconds to 3.22 seconds. The variation recorded is 0.22 seconds. When we compare sprint values, the experiment group has a statistically significant advantage over the control group (p<0.05). In the control group, 20-meter hurdle sprint score decreased from 3.87 seconds to 3.79 seconds. Total variation recorded is 0.08 seconds. And in the experimental group, the 20-meter hurdling score decreased to 3.60 seconds from 3.75 seconds. The total variation recorded is 0.15 seconds. Comparing the difference in hurdle race time between two groups is not statistically significant. (p> 0.05). The horizontal jump distance in the control group increased to 268.80 cm from 266.93 cm. The score increased from 261.13 cm to 267.80 cm in the experimental group. There is an increase of 6.67 cm. When comparing the horizontal jump values, there is statistically significant difference on the experiment group over the control group (p <0.05).

***Keywords:*** *chiropractic, manipulation, splash, sprint, HVLA, sacroiliac, soccer player, lumbosacral*

### P-17 Assessment of immediate effects of sacroiliac joint manipulation on hamstring muscle strength

#### Mehmet Toprak^1^, Dilber Coskunsu^1^, Doruk Turhan^2^, Resat Coskun^3^

##### ^1^Bahcesehir University, Istanbul, Turkey; ^2^Doruk Turhan, PT, Lecturer, Altinbas University, Istanbul, Turkey; ^3^Resat Coskun, PT, Lecturer, Arel University, Istanbul, Turkey

###### **Correspondence:** Mehmet Toprak

**Study objectives**

The two forces arising from body weight and compression of the hip joint forming a nutation torque in the sacroillac joint which result in a moment arm moving through the axis of rotation. This moment transmits a force through Biceps Femoris muscle and Sacrotuberous ligament along the sacroilliac joint line by muscle contraction. Thus, a correct joint angle allows the most efficient muscle contraction without loss of strength along the moment arm.

The aim of this study was to investigate the immediate effect of chiropractic (HVLA) Sacroiliac Joint manipulation on hamstring muscle strength in healthy individuals with asymptomatic Sacroiliac joints dysfunction.

**Material and methods**

A total of 60 individuals were included in the study. The participants were selected in the ages between 18- 40. In the practice group, two groups were separated as 40 individuals, and individual selection was randomized.

Leg lengths of the healthy subjects will be investigated by performing “Thompson Leg Check Analysis” on healthy subjects. After this analysis, participants who have functional shortness in leg length were subjected to a corrective HVLA thrust manipulation through sacroiliac joint. No application was made to the control group. One-time chiropractic HVLA sacroiliac manipulation was applied to the experiment group. Hamstring muscle strengths assessment were made before and after the application in healthy subjects. MicroFet dynamometer was used for assessment of muscle strength.

**Results**

Statistical analysis shows that in the control group; initial assessment of the right hamstring strength was recorded as 41.77 kg and the final assessment was 38.28 kg. There was a decrease of 3.49 kg (p> 0.05). First leg evaluation of left hamstring strength 59,34 kg and the final assessment recorded was 41.96 kg. There was a decrease of 17.38 kg (p> 0.05). In the experiment group, the initial assessment of the right hamstring strength is recorded as 34.68 kg while final evaluation was recorded as 43.29 kg. An increase of 8.61 kg was recorded and found statistically significant (p <0.05). The initial evaluation of the left leg hamstring strength was 35.96 kg while final evaluation was recorded as 43.64 kg. An increase of 7.68 kg was recorded and found statistically significant (p <0.05).

***Keywords:*** *chiropractic, manipulation, HVLA, sacroilliac, lumbosacral, hamstring*

### P-18 Neck function in male rugby league athletes: a comparison of age and professionalism

#### Bianca Zietsman, Fatma Bosnina, Niall Tilley, Pia Helminen, Daniel Morgan, Peter McCarthy

##### University of South Wales, Pontypridd, UK

###### **Correspondence:** Bianca Zietsman

**Introduction**

Rugby is one of the most popular contact sports known worldwide, played by both males and females of all ages. Since it is a complex and high demanding sport, injury rates can be significant. The most common rugby league injuries appear to be those to the head and neck. Active Cervical Spine Range of Motion (ACRoM) has been shown to decrease following acute trauma and repeated low grade injuries.

This study aims to compare the neck function in terms of ACRoM between male rugby league athletes at different levels of professionalism and age.

**Methods and material**

We used secondary data analysis of data gathered previously to compare ACRoM of 43 professional rugby league players (Twenty one from the National Australian Rugby League Team: mean age 27.05 years, s=3.08; Twenty two from the fully professional London Broncos team: mean age 24.4 years, s=4.24), Fifteen semi-professional players from South Wales Ironmen (mean age 25.4, s=4.93), and Fourteen junior rugby league players from colleg y cymoed (mean age 18, s=1). ACRoM in Flexion-extension (sagittal) coupled movements were chosen to be measured using a cervical range of motion device (CROM). This ethical approval was granted by the faculty of life sciences and education ethics committee of the University of South Wales.

**Results**

There appeared to be no correlation between players’ age and their ACRoM. There was a significant difference between the three levels in terms of flexion and total sagittal movements (Flexion: 57.65±13.65; 49.20±15.83; 65.73±12.22; *P* = 0.007, total sagittal: 116±18.7;111.13±18.72; 128.20 ± 19.81; *P* = 0.045, for professional, semiprofessional, Junior in both ranges, respectively). However, there was no difference between the extension movement ranges (59.26±9.93;61.93±16.52; 62.47 ± 12.63;*P* = 0.589, for professional, semiprofessional, Junior, respectively).

**Conclusion**

These results suggest that although there was no correlation between sagittal ACRoM and age, junior league players have the highest sagittal ACRoM, while the semi-professionals have the lowest.

### P-19 Elite male athletes: comparative study of neck function (ACROM)

#### Bianca Zietsman, Chirstopher Bagsworth, Niall Tilley, Pia Helminen, Daniel Morgan, Peter McCarthy

##### University of South Wales, Pontypridd, UK

###### **Correspondence:** Bianca Zietsman

Although elite athletes performance is highly monitored and controlled, there are aspects of elite level performance which can be ignored^1^. One area of relative neglect we have reported on previously is that of cervical spine function^2^. This area is pivotal in head positioning and as such even minimal dysfunction might compromise performance. Here we report variations in cervical spine function across a range of elite male athletes.

Protocol used is the same as that described previously in Lark and McCarthy, 2007.

A cervical range of motion device (CROM)^3^ was used to measure ACROM following a warm up procedure. Ethical approval was granted by The Faculty of HSS Ethics Committee, University of Glamorgan, written informed consent was obtained from all subjects.

These findings confirm that playing elite contact sport such as rugby, both union and league and ice hockey is associated with a large decrease in ACROM. This suggests that these groups have a similar risk of degeneration to the vertebral joints as 60 year old “normals”. Helmet wearing sports such as American Football and Ice Hockey appear to have an altered head position to the groups. Research into preventative methods needs to be considered.

